# Automated and personalized glioblastoma tumor organoid drug screening platform exposes sensitivity to proteasome and HDAC inhibitors

**DOI:** 10.1038/s41698-026-01552-5

**Published:** 2026-06-16

**Authors:** Gerhard Jungwirth, Adrian Paul, Amélie Wöllner, Rolf Warta, Junguo Cao, Valentina Fermi, Lena Jassowicz, Philip Dao Trong, Andreas von Deimling, Juergen Debus, Sandro Krieg, Andreas Unterberg, Amir Abdollahi, Christel Herold-Mende

**Affiliations:** 1 Division of Experimental Neurosurgery, Department of Neurosurgery, Medical Faculty of Heidelberg, Heidelberg University, Heidelberg, Germany; 2 German Cancer Consortium (DKTK) Core-Center Heidelberg, Heidelberg Institute of Radiation Oncology (HIRO), National Center for Radiation Oncology (NCRO), German Cancer Research Center (DKFZ), Heidelberg, Germany; 3 Department of Neuropathology, Institute of Pathology, Heidelberg University, Heidelberg, Germany; 4 Division of Molecular and Translational Radiation Oncology, Heidelberg Faculty of Medicine (MFHD), Heidelberg University Hospital (UKHD), Heidelberg Ion-Beam Therapy Center (HIT), Heidelberg, Germany; 5 Clinical Cooperation Unit Translational Radiation Oncology, National Center for Tumor Diseases (NCT), Heidelberg University Hospital (UKHD) and German Cancer Research Center (DKFZ), Heidelberg, Germany

**Keywords:** Cancer, Drug discovery, Oncology

## Abstract

Personalized drug screening aims to improve the survival of glioblastoma (GBM) patients by identifying effective patient-individual drugs. Therefore, we conducted an automated high-throughput drug screening (aHTS) on standardized patient-derived glioblastoma tumor organoids (TOs). Robot-assisted aHTS was performed on TOs from 11 GBM patients. TOs fully compacted after two days, and the size remained stable over 10 days. TOs proliferated over time, and immunofluorescence stainings (GFAP, Tenascin C) confirmed tissue-like architecture. Anti-glioma effects with the lowest drug concentrations were achieved for proteasome inhibitors (carfilzomib, bortezomib, ixazomib), and HDAC inhibitors (panobinostat, romidepsin). Plasma *C*_max_-based drug levels (*C*_max_/IC_50_ > 1) used as a surrogate were only achieved for the three proteasome inhibitors and the HDACi romidepsin. The impact of their drug targets PSMB5 and HDAC1/2 on the growth of GBM cells was successfully validated by RNAi experiments. We established an aHTS platform for GBM TOs, and identified proteasome and HDAC inhibitors as promising drugs for the treatment of GBMs.

## Introduction

Glioblastoma (GBM) remains one of the most common and deadliest tumors to date. Standard treatment in newly diagnosed patients consists of maximal safe resection followed by radiotherapy and concomitant temozolomide treatment (TMZ)^1^. Still, median progression-free survival (PFS) is merely 6–7 months, and median overall survival (OS) is limited to only 12–18 months^1^.

There is a large body of evidence that intrinsic drug resistance as well as molecular heterogeneity contribute to the lack of effectiveness in numerous clinical trials^2–4^. GBMs contain multiple and diverse genetic aberrations in receptor tyrosine kinases (RTK), p53, and other pathways that may be addressed by targeted therapies^5,6^. However, in unselected patient cohorts, targeted intervention against key recurrent oncogenes in the RTK pathways, EGFR and PDGFRA, failed to improve overall survival^4,7^. In search for more effective therapies, a few preclinical studies tested larger numbers of drugs. However, most drug response data were obtained on GBM cell lines or glioma stem-like cells, lacking the impact of the tumor microenvironment^8–11^. In one of these studies, screening of 132 drugs in 27 patient-derived glioma lines identified that neuroactive drugs such as the antidepressant vortioxetine demonstrated potent anti-glioblastoma effects in combination with the current standard-of-care therapies in vitro and in vivo^12^.

Patient-derived tumor organoids (TOs) are mini-tumors generated from tumor tissues within a few days, which, under optimal conditions preserve the parental geno- and phenotype and maintain the cellular heterogeneity and key components of the tumor microenvironment^13,14^. This exciting new technology provides representative avatars of the patient tumor in large numbers and thus can be exploited for drug discovery and drug screening purposes more efficiently than conventional cell lines or syngeneic or immunocompromised xenografted mouse models^15^. In combination with molecular profiling of the tumor, there is some hope that treatment sensitivity data of TOs will guide clinical treatment decisions in the future^13^. This assumption is reinforced by a recent review summarizing drug screening data on patient-derived organoids and their ability to predict patients’ treatment responses^16^. Importantly, in most of the studies, the selection of drugs based on an organoid-based drug screening was associated with an improved clinical response to a given treatment^16^. This underlines the strength of patient-derived tumor organoids as an important model for predicting drug responses on a patient-individual level. However, regarding GBM TOs, there are only proof-of-concept studies testing a small number of drugs in small patient cohorts^15,17^. Therefore, larger drug libraries and patient numbers are required to fully explore the pharmacological landscape of GBM tumor organoids to provide every patient with a suitable treatment recommendation.

In the current study, we established an automated high-throughput drug screening platform based on standardized glioblastoma tumor organoids and performed a drug screening of 166 FDA-approved oncology drugs within eight days after surgery. This personalized drug screening provides novel insight into the pharmacological landscape of GBMs by revealing sensitivities to proteasome, HDAC, and RNA/protein-translation inhibitors.

## Results

### Establishment of a standardized patient-derived tumor organoid model

**Fig. 1:**
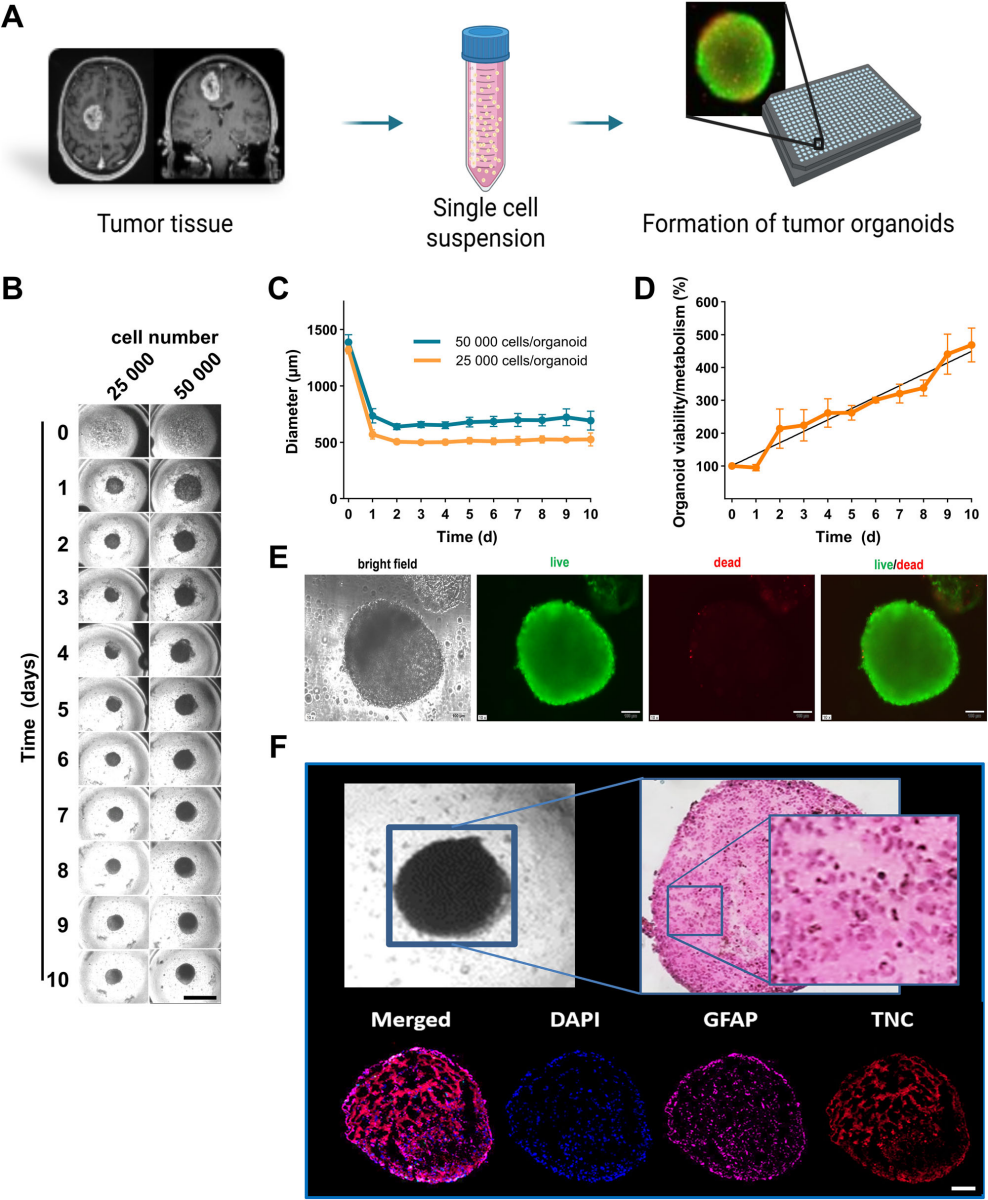
Preparation and formation of GBM tumor organoids. **A** Freshly resected surgical GBM specimens were used to prepare single cell suspensions. Defined numbers of single cells were seeded into anti-adhesive 384-well plates. **B** GBM TOs reaggregated within one to two days and maintained their size over 10 days. Bar represents 1 mm. **C** GBM TOs from six patients (*n* = 6) were generated at 25,000 or 50,000 cells per organoid and monitored for 10 days. The TO diameter (µm) was assessed microscopically. **D** Metabolic activity/viability of GBM TOs from three patients (*n* = 3) was measured daily. ATP levels linearly increased within the 10-day observation period, reflecting the proliferation and viability of GBM TOs. Data are presented as mean ± SEM. **E** The bright field image and live (green)/dead (red) stainings demonstrate a vital tumor organoid generated from a primary GBM on day 9. Bar represents 100 µm. **F** Brightfield pictures of entire TO, HE stainings of TO-derived cryosections, and multicolor immunofluorescent stainings including a nuclear staining (DAPI), a staining of the lineage marker glial fibrillary acidic protein (GFAP), and the extracellular matrix protein Tenascin C (TNC), confirming the successful establishment of TOs with a tissue-like organization. Bar represents 100 µm. Data in (**C**) and (**D**) are depicted as mean ± SEM.

To enable large-scale personalized drug screenings, we first developed a protocol to generate standardized glioblastoma tumor organoids (TOs) in large quantities (Fig. 1A). To ensure high success rates of TO generation, we only used high-quality tissue samples from the contrast-enhancing part of the tumor while simultaneously avoiding necrotic pieces. In general, single cells re-aggregated to form TOs within one to two days resulting in a reduced diameter by at least 60% independent of the seeded cell number (Fig. 1B, C). The TO size remained comparable among the biological replicates and morphologically stable throughout a 10-days observation period. The metabolic activity increased steadily over time as assessed by ATP-based CellTiter-Glo 3D (Fig. 1D). TOs mainly consisted of viable cells, while dead cells were predominantly found outside of the organoid as evidenced by live/dead staining (Fig. 1E). To illustrate that our GBM TOs resemble the parental tumor, we stained TOs for lineage markers (GFAP) expressed and matrix proteins known to be enriched in the tumor tissue of GBMs (tenascin C, TNC, Fig. 1F). Expression of glial fibrillary acid protein (GFAP) was observed in the majority of cells indicating the presence of tumor cells. Moreover, we observed a strong expression of the extracellular matrix protein Tenascin C (TNC), which is one of the most highly upregulated genes in GBM^18^. Taken together, we established a protocol to form standardized TOs from GBMs, which can be used for personalized drug screening.

### Implementation of an automated high-throughput drug screening platform

**Fig. 2:**
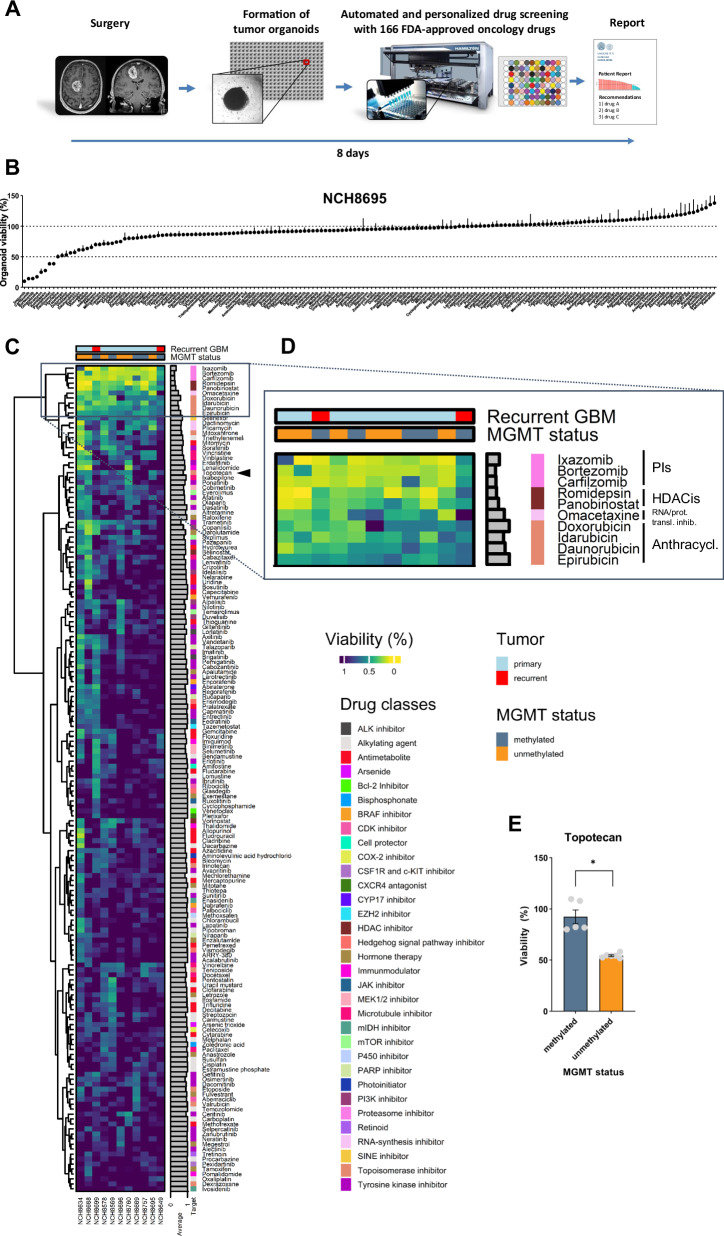
Automated high-throughput drug screening (aHTS) in GBM tumor organoids. **A** Workflow: From GBM-derived single cell suspensions, 924 TOs were generated on three anti-adhesive 384-well plates. The aHTS was performed by the liquid handling robot Hamilton MicroLAB STAR®. The drug library NCI AODX consisted of 166 oncology drugs. The screening was done in a two-step approach. First, drugs were screened in a single concentration (2.5 µM) in triplicate. Then, the top five most effective drugs were selected to treat TOs from the third 384-well plate in semi-logarithmic concentrations from 3 nM to 10 µM. **B** Drug sensitivity data of a representative patient (NCH8695). Data are depicted as mean ± SEM. **C** Heatmap summarizing drug sensitivity data of 11 IDHwt GBM patients, including two recurrent tumors. The black arrow indicates topotecan. **D** Three drug classes demonstrated the highest drug sensitivity at 2.5 µM, including proteasome inhibitors (bortezomib, carfilzomib, and ixazomib), HDAC inhibitors (romidepsin, panobinostat), topoisomerase inhibitors (doxorubicin, idarubicin, daunorubicin, epirubicin), and RNA/protein translation inhibitor omacetaxine. **E** Vulnerability stratified for MGMT methylation status revealed differential drug responses of topotecan-treated organoids. The *P-*value was calculated using an unpaired Student’s t-test, corrected for multiple comparisons using the Holm-Sidak method. *P*-values < 0.05 were considered significant (*P < .05).

As a next step, we developed an automated high-throughput drug screening platform (Fig. 2A). After the preparation of tumor organoids from freshly prepared tumor tissue, we screened an antineoplastic drug library of the National Cancer Institute (AODX) on TOs from nine patients suffering from newly diagnosed GBM and two recurrent GBMs. Included were patients with radiological suspicion of newly diagnosed glioblastoma as well as patients with histologically confirmed recurrent glioblastoma from which sufficient material for subsequent drug screening was available. The patient’s clinicopathological characteristics are presented in Table 1. Drug screening was conducted in two steps: First, 166 FDA-approved drugs were tested at a single concentration of 2.5 µM in triplicate for 72 h, and viability was assessed by CellTiter-Glo 3D. Drug screening concentration was selected to be in the range of the calculated median peak serum concentration (*C*_max_) of all employed drugs (median *C*_max_: 1.9 µM, interquartile range: range 0.46–9.26, Supplementary Fig. 1A). A summary of *C*_max_ values for this drug library has recently been published^19,20^. Missing information was complemented using the available data from the NIH Inxight Drugs program^21^. For the five drugs with the lowest viability values from each case, we further prepared dose-response curves (DRCs) on an additional 384-well plate. By using this two-step protocol, final results from the drug screening could be generated within eight days after surgery.

**Table 1:** Clinicopathological information of GBM patients

Clinical Parameters		Patients	
		***n***	**(%)**
**Sex**	Male	7	63.6
	Female	4	36.4
**Age at surgery (years)**	Median	63	
	Range	40-78	
**Primary or recurrent tumor**	Primary	9	81.8
	Recurrent	2	18.2
**GBM Subtype**	RTK I	3	27.3
	RTK II	2	18.2
	mesenchymal	1	9.0
	NA	5	45.5
**MGMT status**	Methylated	5	45.5
	Unmethylated	6	54.5
**Extent of resection**	Total	5	45.5
	Subtotal	6	54.5
**Postoperative treatment**	Radiotherapy	4	36.4
	Chemotherapy	0	0
	Radiochemotherapy	7	63.6

This large-scale automated drug screening revealed that only a small fraction of drugs reduced TO’s viability to or below 50% (0.048%, *n* = 8/166) across all patients (*n* = 11) with substantial differences among single cases (Fig. 2B, C). The top ten best-performing drugs in each patient summed up to 35 compounds targeting 14 different modes of action. The most sensitive drugs were bortezomib (21.8% average viability, range 14.2–49.7), ixazomib (24.1%, 7.4–74.4), carfilzomib (24.2%,12.9–68.6), romidepsin (24.9%, 6.4–71.0), panobinostat (31.5%, 10.2–57.8), idarubicin (41.8%, 23.4–71.4), omacetaxine (47.8%,17.1–103.3), and daunorubicin (47.8%, 17.4–74.1). Interestingly, hierarchical clustering resulted in groupings of drugs with similar modes of action, providing evidence for a drug class-specific vulnerability (Fig. 2D). The most sensitive drug group consisted of PIs (proteasome inhibitors): ixazomib, carfilzomib, and bortezomib. The second cluster contained drugs targeting epigenetic regulation, panobinostat, and romidepsin. The third group comprised topoisomerase inhibitors, including doxorubicin, idarubicin, daunorubicin, and epirubicin. No differences in drug responses were observed between primary and recurrent GBMs, but this finding may be limited by the small sample size of recurrent tumors (*n* = 9 vs *n* = 2; Supplementary Fig. 1B). Furthermore, when stratifying for the MGMT methylation status, only the topoisomerase inhibitor topotecan demonstrated remarkably higher sensitivity in unmethylated GBMs (54.3% vs 99.8% viability, *P*_adj_ = 0.021, Fig. 2E, Supplementary Fig. 1C, D). In conclusion, the pharmacological landscape of GBMs revealed ineffectiveness to most drug classes within the tested concentration range, except for proteasome, HDAC, and topoisomerase inhibitors.

### Favorable *C*_max_/IC_50_ ratios prioritize proteasome inhibitors and HDACi romidepsin

**Fig. 3:**
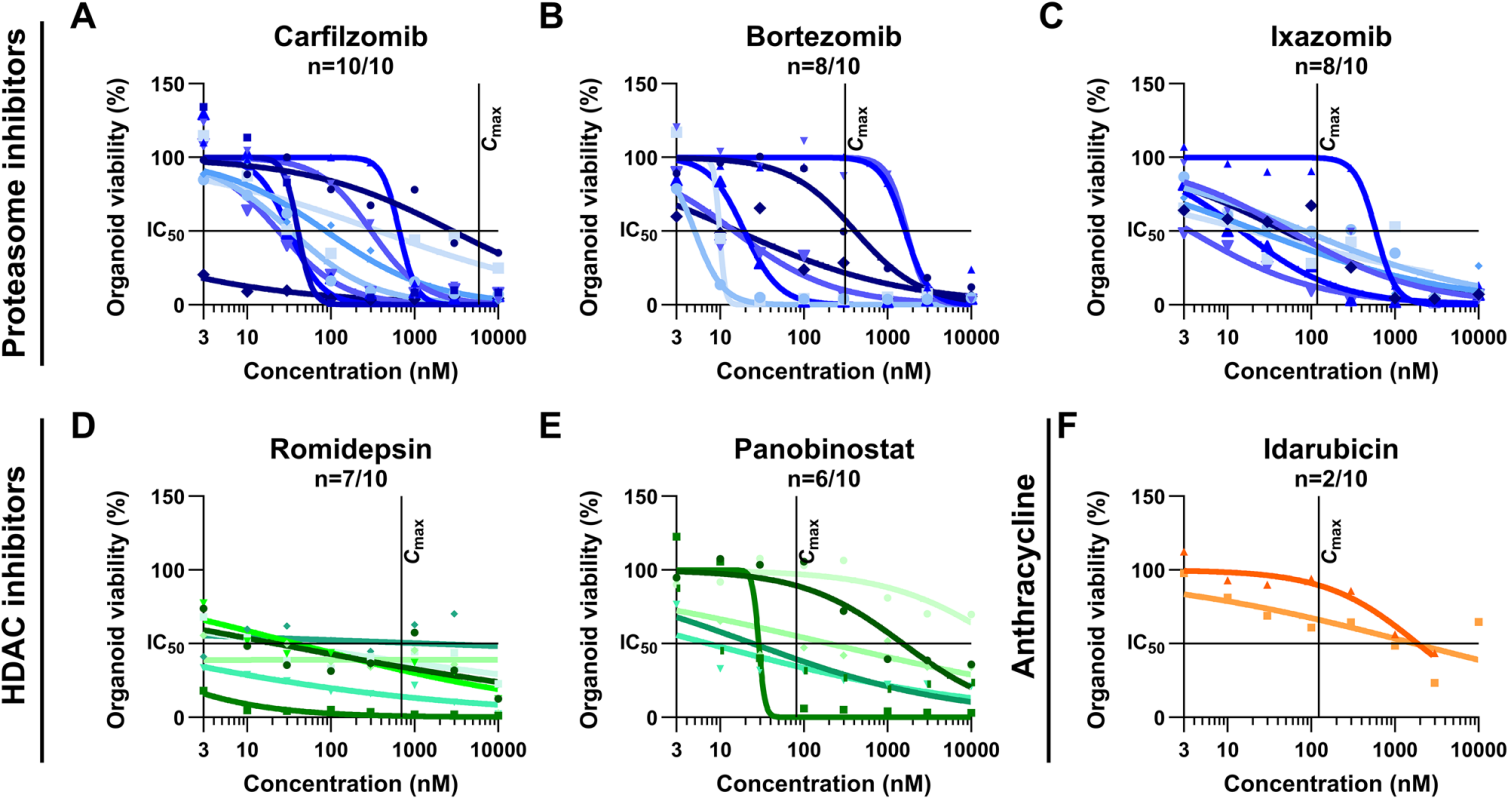
Proteasome and HDAC inhibitors most frequently show the highest drug sensitivity. **A**–**C** Dose-response curves (DRCs) were generated if the drug was among the most sensitive drugs in the first screening step. In almost all cases, the proteasome inhibitors carfilzomib, bortezomib, and ixazomib belonged to the most sensitive drugs. *C*_*max*_ indicates the peak serum concentration of the respective drug. **D**, **E** Dose-response curves for the HDAC inhibitors romidepsin and panobinostat. **F** DRCs for the anthracycline idarubicin are depicted.

To further explore the efficacy of the top five drugs from each case, DRCs were performed in tumor organoids, and the half-maximal inhibitory concentrations (IC_50s_) were calculated (Fig. 3*;* Supplementary Table S1). Only in one case, the preparation of DRCs was not possible due to insufficient cell numbers. Using this approach, the PI carfilzomib has been in all 10 cases among the most effective drugs. Bortezomib and ixazomib had been effective in 80% of the patients. DRCs from HDACis romidepsin and panobinostat were conducted in 70% and 60% of the cases. In contrast, the anthracycline idarubicin has only been found in two patients to be among the most effective drugs. In these cases, the PIs displayed similar average IC_50_ values. The average IC_50_ value of carfilzomib was 91 nM (51–168 95%CI), bortezomib exhibited 64 nM (25–153), and ixazomib 38 nM (20–68) (Fig. 3A–C). Treatment with HDAC inhibitors resulted in IC_50_ values of <3 nM for romidepsin (0.01–14) and 259 nM for panobinostat (77–049) (Fig. 3D, E). The anthracycline idarubicin had the highest average IC_50_ value of 2.2 µM (0.6–31) (Fig. 3F). We next examined whether MGMT methylation status influenced drug sensitivity by comparing IC_50_ values between methylated and unmethylated tumors. MGMT methylation status was not associated with significant differences in sensitivity to carfilzomib, bortezomib, ixazomib, romidepsin, or panobinostat (Supplementary Fig. 2).

Next, we compared IC_50_ values to the respective drugs’ peak serum concentrations (*C*_max_) (Table 2). The ratio of *C*_max_/IC_50_ might indicate a favorable systemic drug exposure relative to the ex vivo potency (>1)^20^. By using this approach, favorable *C*_max_/IC_50_ ratios seem to be achievable for the PIs carfilzomib (ratio 64.62x), and to a much lesser extent bortezomib (4.88x), and ixazomib (3.11x). Furthermore, the HDACi romidepsin (232.33x) also seems to reach a favorable *C*_max_/IC_50_ ratio. In contrast, panobinostat’s and idarubicin’s drug levels might not be sufficient (0.32x, and 0.05x). These data indicate that selected proteasome and HDAC inhibitors should be prioritized for further investigation.

**Table 2:** Half-maximal inhibitory concentrations (IC_50_), peak serum concentrations, and the *C*_max_/IC_50_ ratio for the top sensitive drugs

Drug	IC_50_ (95% CI) [nM]	*C*_max_ [nM]	*C*_max_/IC_50_ ratio
Carfilzomib	91 (51–168)	5880	64.62
Bortezomib	64 (25–153)	312	4.88
Ixazomib	38 (20–68)	118	3.11
Romidepsin	<3 (0.01–14)	697	232.33
Panobinostat	259 (77–1049)	82	0.32
Idarubicin	2296 (672–31708)	123	0.05

### Target validation confirms therapeutic vulnerabilities to PSMB5 and HDAC1/2 inhibition

**Fig. 4:**
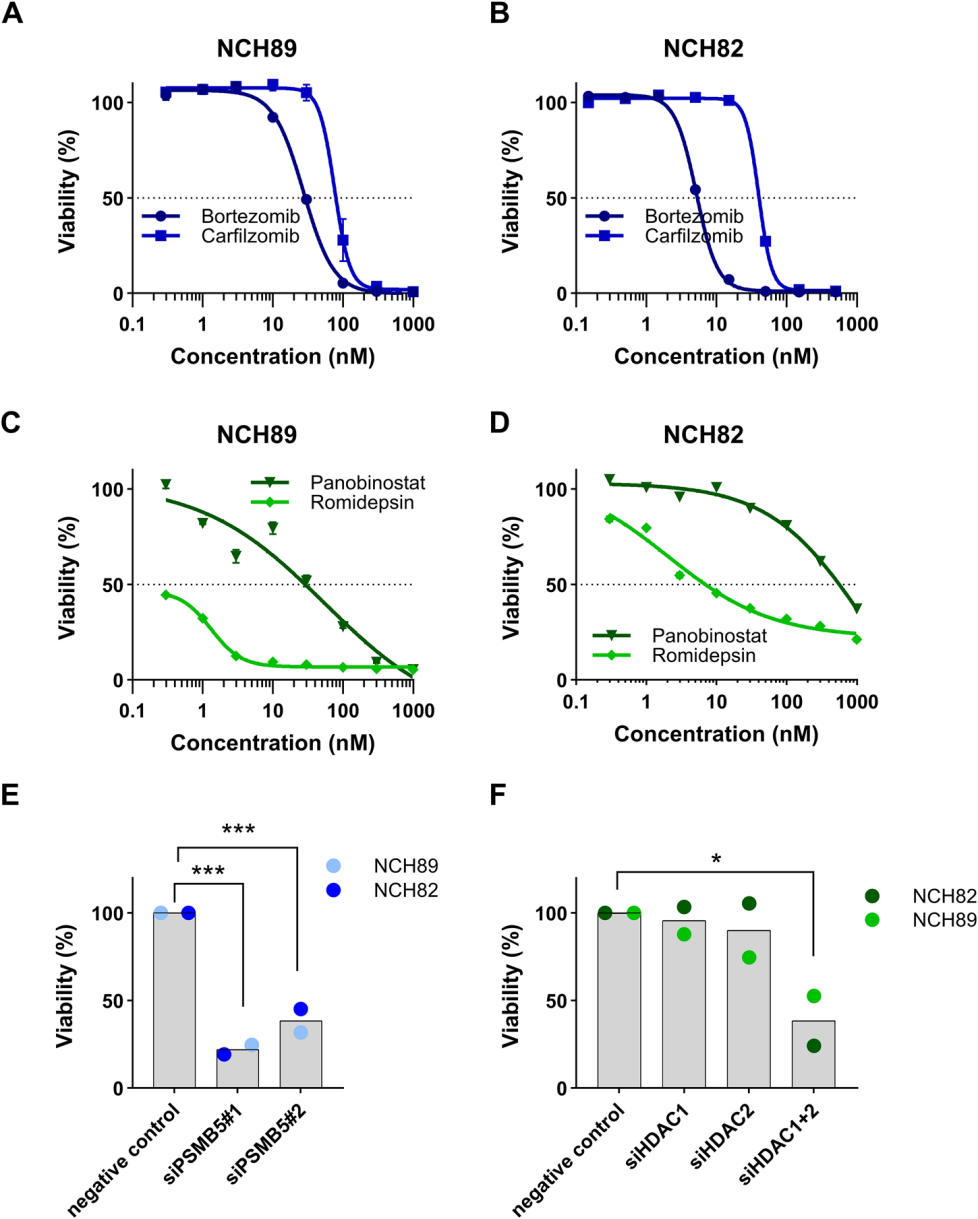
Proteasome and HDAC inhibitor targets are essential for the growth of GBM cells. **A**, **B** Dose-response curves (DRCs) for the proteasome inhibitors bortezomib and carfilzomib in glioblastoma cell lines NCH89 and NCH82. **C**, **D** DRCs of the HDAC inhibitors romidepsin and panobinostat in NCH89 and NCH82 cells. **E** RNAi-mediated knockdown of the proteasome inhibitor target *PSMB5* with two different siRNAs demonstrated significant inhibition of proliferation in vitro. **F** Only double RNAi-mediated knockdown of *HDAC1* or *HDAC2* inhibited proliferation in both cell lines. Data is presented as mean + SEM. *P*-values were calculated using Dunnet’s one-way ANOVA. *P*-values < 0.05 were considered significant (*P < 0.05; **P < 0.01; ***P < 0.001).

As a next step, we sought to study the targets of the most sensitive drug classes. While proteasome and HDAC inhibitors have RNAi-targetable proteins, anthracyclines intercalate with DNA and are therefore not suitable for knockdown experiments^22^. RNAi-mediated knockdowns were conducted in glioblastoma cell lines (NCH82 and NCH89^23^). First, we assessed if these two cell lines were adequate models by showing similar drug responses. Therefore, we prepared dose-response curves for the proteasome and HDAC inhibitors of interest. The average IC_50_ values for carfilzomib and bortezomib exhibited 57 (range: 39–75) and 16 (5–27) nM in both cell lines (Fig. 4A, B). The IC_50_ values for romidepsin and panobinostat were 1.65 (1.3–2) and 647 (54-1240) nM (Fig. 4C, D, Supplementary Fig. 3A), corroborating the suitability of these GBM cell lines for further RNAi experiments. PIs primarily target PSMB5, one important key player of the proteasome complex^24,25^. PSMB5 depletion by two different RNAis revealed a significant decrease in cell viability of both cell lines tested (Fig. 4E). Successful knockdown of the target gene was evaluated by qRT-PCR (Supplementary Fig. 3A, B).

The HDAC family contains 18 genes divided into four classes and two subclasses^26^. Panobinostat is a pan-HDAC inhibitor, while romidepsin specifically targets HDAC1/2^27^. Therefore, we focused our RNAi experiments on the depletion of HDAC1 and 2. Interestingly, HDAC1 or HDAC2 single knockdown had no effect on the cell lines; however, double knockdown significantly decreased the viability (Fig. 4F).

In summary, depletion of the proteasome inhibitor target PSMB5 and the HDACi target HDAC1 and 2 decreases GBM viability in vitro, which is in line with the observed antineoplastic effects of proteasome and HDAC inhibitors.

### Marizomib shows limited ex vivo potency in most GBM organoids

**Fig. 5:**
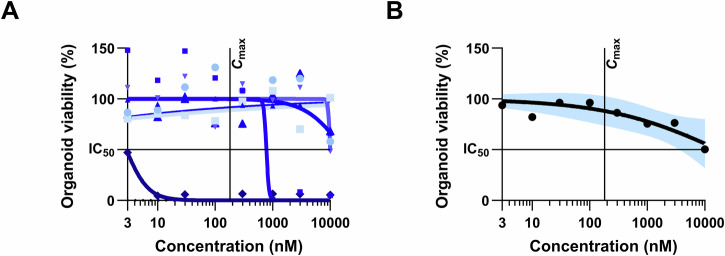
The blood-brain barrier-penetrating proteasome inhibitor marizomib is mainly ineffective in GBM organoids. **A** Dose-response curves (DRCs) of the proteasome inhibitor marizomib in TOs from seven GBM patients. *C*_*max*_ indicates the peak serum concentration of the respective drug. **B** Combined dose-response curves of all patients to estimate the overall sensitivity of the drug. Data is presented in mean+95% confidence interval.

Our data show that PIs exhibit a strong anti-glioma effect in vitro and ex vivo. However, previous studies indicated that carfilzomib does not penetrate the blood-brain barrier sufficiently and that bortezomib reaches only 5–7% of its serum concentrations in animal brains^28,29^. Marizomib has been described as a novel, irreversible, and brain-penetrant pan-PI reaching approximately 30% of its serum drug level in the CNS^30,31^. To explore the efficacy of marizomib in our model, we tested the drug on TOs from a subset of the GBM patients (n = 7). However, marizomib has been ineffective in the majority of cases compared to the other PIs by exhibiting combined IC_50_ values of >10 µM (Fig. 5A, B). The *C*_max_ of the drug is estimated to be up to 182 nM^32^, which translates to a conservatively calculated *C*_max_/IC_50_ ratio of 0.018, and only 0.005 considering the 30% BBB penetration from preclinical studies^31^. Only in one case, the calculated IC_50_ value was <3 nM, translating to a C_max_/IC_50_ ratio of 60.6 and 18.2 when considering the BBB penetration. The drug has been tested in a phase 3 study with newly diagnosed glioblastoma in addition to the standard treatment of temozolomide and radiation^33,34^. In line with our findings, adding marizomib to standard therapy did not improve the OS or PFS of patients^34^. These findings suggest that insufficient ex vivo potency relative to achievable systemic exposure may have contributed to the limited clinical activity of marizomib observed in EORTC-1709, although this remains hypothetical given that our TO model does not recapitulate blood-brain barrier physiology or fully capture intratumoral pharmacokinetics.

## Discussion

Treatment options for glioblastoma patients are still limited. Current therapies encompass surgery, radiation, temozolomide, and tumor-treating fields with only a modest survival rate of 12 to 18 months^1^. Targeted therapies and personalized medicine approaches may enable more precise and effective treatment strategies. Here, we developed a high-throughput platform to screen FDA-approved oncology drugs in patient-derived glioblastoma organoids, followed by an in-depth dose-response characterization of the top drugs. Thereby, we identified GBM sensitivities to a distinct group of proteasome, HDAC, and topoisomerase inhibitors. To evaluate the potential clinical efficacy of the drugs, effective drug concentrations were compared to peak serum levels. Using this approach, only the proteasome inhibitors carfilzomib, bortezomib, and ixazomib, and the HDACi romidepsin showed favorable C_max_/IC_50_ ratios. However, preclinical data suggest that bortezomib and carfilzomib barely penetrate the blood-brain barrier. Testing the newly developed and clinically studied BBB-penetrating proteasome inhibitor marizomib as a replacement, unfortunately, failed because glioblastoma TOs were surprisingly >100x less sensitive to marizomib than the other proteasome inhibitors and did not reach favorable *C*_max_/IC_50_ ratios except in one case. This might provide a possible explanation for the failure of the recent EORTC-1709 trial, and indicates the suitability of GBM TOs as a preclinical model to test treatment responses.

Large-scale drug screens in patient-derived glioblastoma organoids are still lacking, because most preclinical studies focused on cell lines^8,11,12,35^. In one of the largest studies, Johansson et al. explored 1 544 potential cancer and non-cancer drugs in a multistage process, initially few and for a final selection of 94 drugs in a larger number of GBM cell lines, suggesting that a subgroup of GBM lines is more sensitive to proteasome inhibitors^8^. Another stepwise chemogenomic screening was performed in glioma cell lines, detecting among other drug classes highly effective HDACi, including panobinostat and vorinostat^35^, corroborating the by us observed vulnerability towards HDAC inhibition.

PIs block the degradation of intracellular proteins and therefore impair the removal of damaged, misfolded, and short-lived proteins from the cell^36^. This becomes particularly important in the context of cancer, where tumor cells proliferate faster compared to their non-malignant counterparts, and therefore the translated proteins have a higher chance for synthesis errors or oxidative damage^36^. Furthermore, unregulated proteasome activity can lead to increased degradation of tumor suppressors while stabilizing oncogene products^36^. Upon proteasome inhibition, these proteins accumulate within the cell, impairing the cellular function, resulting in the enrichment of pro-apoptotic proteins such as p53, p73, and BAX, leading to cancer cell death^25,36^. PIs also increase the sensitivity of tumor cells to radiotherapy and are therefore an effective method for chemosensitization^36,37^. However, clinical trials of PIs in glioblastoma revealed mixed results^38–41^. The most promising phase II study added bortezomib to the current standard radiochemotherapy in newly-diagnosed GBM patients and achieved benefits for MGMT methylated GBM patients^41^. However, in our screening, no differential drug responses were observed in TOs of MGMT methylated vs unmethylated patients. Currently available proteasome inhibitors, including carfilzomib or bortezomib, barely penetrate the blood-brain barrier^28,29^. Therefore, PIs with increased BBB permeability were developed to improve their efficacy. Marizomib is such a novel, irreversible, and brain-penetrant pan-proteasome inhibitor that reaches approximately 30% of its serum drug level in the CNS^30,31^. However, the drug in combination with the standard treatment of temozolomide and radiation failed to show any improvement of OS or PFS in the analysis of a phase III study^33,34^. These contradictory results are in line with the low sensitivity observed in our study. While carfilzomib, bortezomib, and ixazomib exhibited low nanomolar IC_50_ of ~64 nM in our GBM TOs, marizomib’s IC_50_ values were almost 200x higher (>10 µM), diminishing marizomib’s advantage of a superior BBB-penetration of 30% compared to bortezomib’s 5-7%^29–31^. Based on our findings, therapeutic drug levels might be achieved in only a small subset of marizomib-treated cases, underscoring the need for personalized drug screening. Taken together, patient-derived tumor organoids may serve as a predictive model for treatment responses in cancer patients^16^.

Protocols to generate TOs vary widely^13–15,17^. One of the most commonly used protocols for generating GBM TOs was reported by Jacob et al. Minced GBM pieces were placed in a medium on an orbital shaker to facilitate organoid generation, which generally formed within 1–2 weeks^17^. The generated TOs resembled their parental tumor regarding protein expression of the glial (tumor) markers glial fibrillary acidic protein (GFAP) and S100β, as well as the stem cell markers Nestin and Olig2, and retained the transcriptomic and genomic features of the primary tumor^17^. However, the evident drawbacks of this protocol are that organoids in this way are not equal in size and viability, differ in cellular composition, and take a prolonged time to establish, all factors that hinder large-scale personalized drug screenings. The protocol presented in this study circumvents these problems by generating standardized TOs in cell number, size, and composition by re-aggregating single cell suspensions. TOs were ready for drug screenings two days after surgery, and the whole drug screening protocol presented in this work can be performed within a reasonable timeframe of 8 days, providing feasible point-of-care testing in a clinical setting and therefore much faster compared to other protocols taking weeks to months to complete the drug testing^17,42^.

A limitation of our study is that the *C*_max_ values are derived from systemic plasma exposure^19–21^ and therefore do not fully capture drug levels in the contrast-enhancing and non-enhancing tumor compartments in glioblastoma^43,44^. Because blood-brain barrier permeability, active efflux transport, and protein binding can substantially reduce CNS and intratumoral exposure, drug concentrations in non-enhancing tumor tissue and in normal brain parenchyma are often lower than plasma^43,44^. In contrast-enhancing tumor tissue, limited data suggest higher tumor-to-plasma ratios for selected compounds, but these data are sparse and not generalizable^43,44^. Consequently, the *C*_max_/IC_50_ ratio should be interpreted as a surrogate parameter, and in this context, a *C*_max_/IC_50_ ratio < 1 may suggest that a compound is less likely to reach effective intratumoral concentrations under standard systemic dosing, and thus may have limited clinical utility in GBM without strategies to improve CNS/tumor exposure. Conversely, even ratios > 1 do not guarantee adequate tumor delivery; however, these candidates should be prioritized for follow-up studies and evaluated in orthotopic GBM models, ideally together with measurements of brain/intratumoral exposure and pharmacodynamic target engagement.

Furthermore, future prospective studies should evaluate whether patient-individual organoid drug responses can guide therapeutic decision-making in GBM, similar to other tumors^45^ and should ideally be combined with matched genomic, transcriptomic, and single-cell profiling of parental tumors and corresponding tumor organoids. Such integrated studies will be required to determine whether specific drug sensitivities are associated with molecular subtypes, transcriptional states, epigenetic programs, differences in cellular composition, and ultimately clinical outcome. In addition, genetic perturbation experiments directly in GBM tumor organoids would represent a valuable mechanistic extension to validate candidate dependencies, assess rescue strategies, and define downstream pathways underlying differential drug sensitivity. In the present study, RNAi-mediated target validation was performed in GBM cell lines because patient-derived material was limited, and reproducible genetic perturbation in short-term TOs, particularly combined HDAC1/2 depletion, requires further optimization.

In summary, we developed a well-standardized high-throughput platform to screen 166 FDA-approved oncology drugs on patient-derived glioblastoma organoids as a next step towards personalized medicine. Finally, our study identified a substantial glioblastoma vulnerability to several proteasome and HDAC inhibitors.

## Methods

### Tumor samples

Human IDHwt glioblastoma specimens were obtained intraoperatively. The use of patient material was in compliance with the Declaration of Helsinki and was approved by the institutional review board (S-005/2003; Medical Faculty Heidelberg). Written informed consent was received from all patients. Patients were included with radiological suspicion of newly diagnosed glioblastoma, as well as patients with histologically confirmed recurrent glioblastoma, and when sufficient viable, non-necrotic tumor tissue from the contrast-enhancing tumor region was available to generate the required cell number for TO formation and drug screening. Samples were excluded when tissue quality was poor, when the material was predominantly necrotic, or when the available tumor volume was insufficient for reproducible organoid generation and downstream screening.

### Establishment of patient-derived glioblastoma tumor organoids

Freshly resected glioblastoma tissue was mechanically and enzymatically dissociated as described before^46^. The generated single cell suspension was incubated in 5 ml Red Blood cell lysis buffer for 5 min at RT. Next, 20 ml PBS was added, and the suspension was centrifuged at 300 *g* for 10 min. The cell pellet was resuspended in a 5 ml cell culture medium. 1.250 to 100.000 cells per well were seeded in the presence of 2% Matrigel Matrix Basement Membrane (Corning, REF #354234) in anti-adhesive 96- or 384-well plates. Plates were centrifuged for 5 min at 500 g to allow the formation of tumor organoids. Images of tumor organoids were taken for 14 consecutive days. The diameter of TOs was measured using ImageJ (1.8.0). The viability of TOs was measured daily using CellTiter-Glo 3D (Promega) and normalized to day 0.

### Automated high-throughput tumor organoid drug screening

Tumor organoids from 11 glioblastoma patients were generated in three anti-adhesive 384-well plates. To ensure the sufficient representation of low-abundance cells 25.000 cells/TO were chosen. Organoid morphology and compaction were monitored microscopically before treatment. On day 2, TOs from two 384-well plates were treated with 166 FDA-approved compounds (National Cancer Institute, AODX) at 2.5 µM in triplicate by the automated liquid handler Hamilton STAR®. Staurosporine, DMSO, and untreated were used as controls on every plate. After 72 hours, equal CellTiter-Glo 3D (Promega) volumes were added to the wells. The plates were then placed on a shaker for 5 min followed by incubation for 25 min at RT. A plate reader (Infinite F200 Pro, Tecan GmbH, Groedig, Austria) recorded luminescence. Data were normalized to DMSO controls. The top five drugs were tested in dose-response curves at 8 concentrations ranging from 3 nM to 10 µM on the third 384-well plate for additional 72 hours. Tumor organoid viability was assessed by CellTiter-Glo 3D (Promega) following the manufacturer’s instructions. The IC_50_ values were calculated using nonlinear regression in GraphPad Prism. Because each patient sample represented a limited surgical specimen, independent repeated drug screening from the same tumor was not feasible.

### Live/Dead staining

Tumor organoids were incubated with an equal volume of the live/dead cell imaging kit (Invitrogen) in each well and incubated for 15 min at RT. Images were taken with a fluorescence microscope (Zeiss, Oberkochen, Germany), and CellSens Dimension software was used for analysis.

### DNA/RNA isolation

Total RNA was extracted from cells using the RNeasy Mini Kit (Qiagen). RNA was quantified by NanoDrop ND-1000 spectrophotometer (Thermo-Scientific, Waltham, MA, USA) and stored at −80 °C until further analysis.

### Immunofluorescence staining

Staining was performed on acetone-fixed cryosections (5-8 µm) of GBM TOs. Anti-GFAP antibody (1:600 dilution, rabbit IgG, Agilent) and anti-Tenascin C antibody (1:25 dilution, mouse IgG, BD Pharmingen) were diluted in DAKO diluent (Agilent). Slides were incubated with primary antibodies at RT for 60 min and washed thrice with PBS-Tween-20 (0.05%). Thereafter, they were incubated for 60 min with the corresponding secondary antibody (1:200 dilution, Invitrogen) diluted in DAPI (1:1000 dilution, Invitrogen) and DPBS (Gibco), followed by three washing steps with PBS-Tween-20. Finally, coverslips were mounted with Elvanol (Sigma-Aldrich). A fluorescence microscope (Zeiss, Oberkochen, Germany) and CellSens Dimension software were used for imaging and analysis.

### Cell lines

The glioblastoma cell lines NCH82^47^ and NCH89^47^ were cultured in Dulbecco’s minimal Eagle’s medium (DMEM, Gibco) supplemented with 10% fetal calf serum (Sigma-Aldrich), 2% L-GlutaMAX (Sigma-Aldrich), and 1% Penicillin/Streptomycin (Sigma-Aldrich) at 37 °C in a humidified environment with 5% CO_2_ atmosphere. Mycoplasma contamination was excluded by 4’,6-diamidino-2-phenylindole staining (Roche, Basel, Switzerland). Cell lines were authenticated by STR DNA profiling analysis (Leibniz Institute DMSZ, Braunschweig, Germany).

### RNAi-mediated knockdown

Glioblastoma cells were transfected with siRNA against *PSMB5* (Cat. #4390824-s11354, #4390824-s11355, Invitrogen), *HDAC1* (Cat. #4427037-s73, 4427038-s74, Invitrogen), *HDAC2* (Cat. #4427037-s6493, 4427038-s6495, Invitrogen) or siRNA negative control (Cat. #4390843, Invitrogen). Lipofectamine RNAiMAX reagent (Cat. #13778030, Invitrogen) was used to deliver siRNAs into the cells. Cells were transfected with a final concentration of 100 nM siRNA and 7.5 µL Lipofectamine RNAiMAX in 1 ml OptiMEM (Gibco) overnight. The next day, the medium was changed to DMEM supplemented with 10% FCS, 2% GlutaMAX (Gibco), and 1% Pen/Strep, and cells were further used for experiments. The knockdown was validated by qRT-PCR.

### Quantitative real-time PCR

Equal amounts of total RNA (1 µg) were reverse-transcribed using the Transcriptor First Strand cDNA Synthesis Kit (Roche) with random hexamer primers for one hour at 50°C. qPCR was performed in triplicate on a LightCycler 480 (Roche) using the LightCycler 480 Probes Master and probes from the Universal Probe Library (Roche) as described [www.roche-applied-science.com]. Relative fold changes between the expression of target genes were calculated using the 2^-ΔΔCq method. *GAPDH*, *ACTB*, and *HPRT1* were used as reference genes. Relative expression levels of *PSMB5*, *HDAC1, and HDAC2* mRNA were normalized to the RNAi control samples. The primers used are shown in Supplementary Table S2.

### Statistical analysis

Data were analyzed using GraphPad Prism 10.0.2 software and the R software environment (4.1). Error bars show the standard error of the mean (SEM) of technical replicates. *P*-values were calculated using a two-tailed Student’s *t*-test or one-way ANOVA. *P*-values < 0.05 were considered significant (* *P* < 0.05; ** *P* < 0.01; *** *P* < 0.001; **** *P* < 0.0001).

## Supplementary information

Supplementary materials.

## Data Availability

The data that support the findings of this study are available in the manuscript and supplementary materials. Further inquiries can be directed to the corresponding authors.
