# Supplementary material for: Automated and personalized glioblastoma tumor organoid drug screening platform exposes sensitivity to proteasome and HDAC inhibitors
Source: NPJ Precis Oncol. 2026 Jun 16;10:228. doi: 10.1038/s41698-026-01552-5 (PMC13273099; doi:10.1038/s41698-026-01552-5)
Supplement: Supplementary file 1 — Supplementary materials. [file 41698_2026_1552_MOESM1_ESM.docx]

**SUPPLEMENTARY FIGURES**


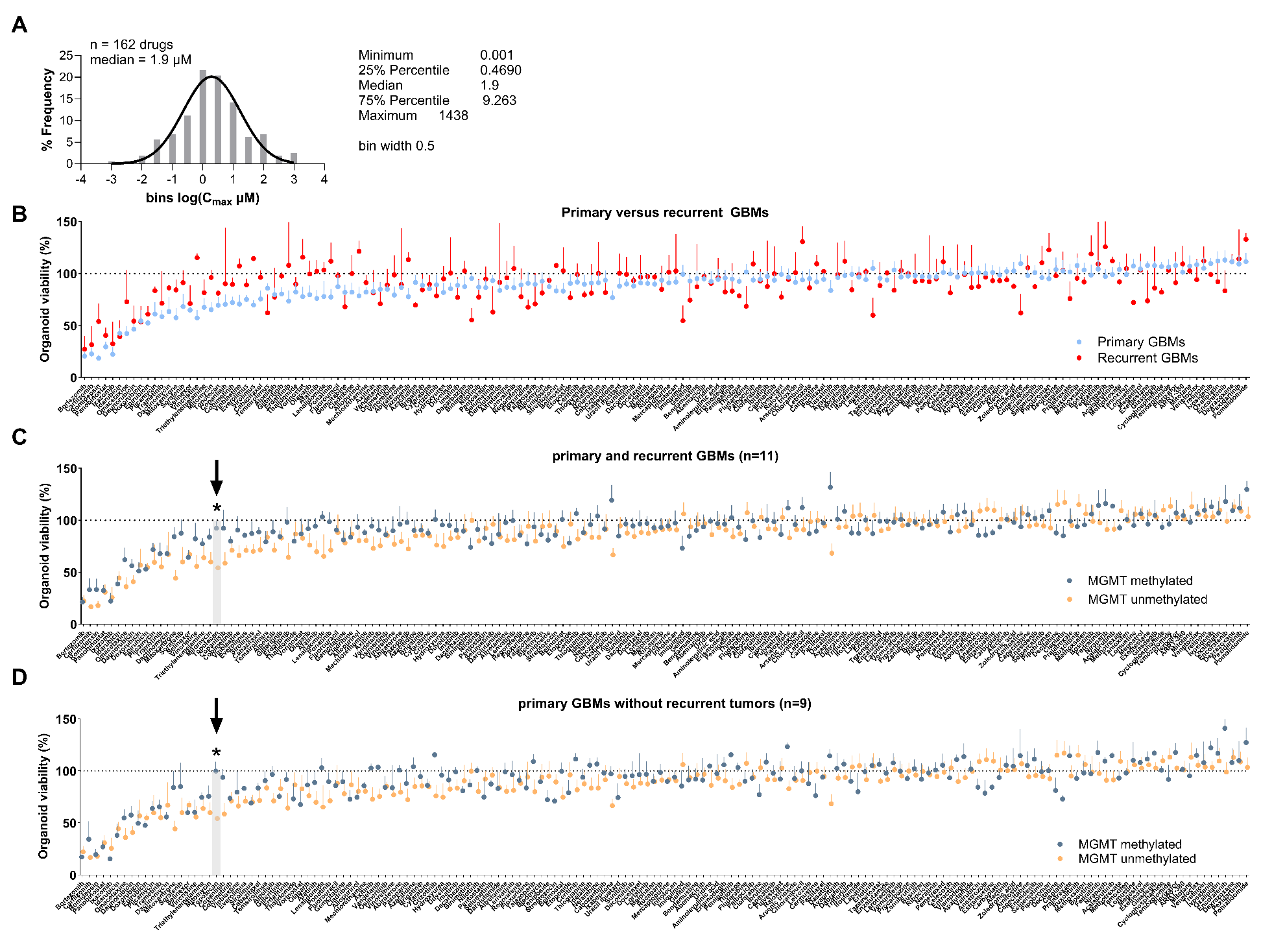


***Supplementary Figure 1 - Analysis of peak serum concentrations and drug responses stratified by primary or recurrent tumors, and MGMT methylation status***

**(A)** Histogram of peak serum concentrations (*C*_max_) of FDA-approved drugs. *C*_max_ data were available for 162 out of 166 drugs. The median *C*_max_ was 1.9 µmol/l. The selected bin width was 0.5. IC_50_ data were derived from recent publications^19,20^ and missing information was complemented using the available data from the NIH Inxight Drugs program^21^. **(B)** Drug responses at 2.5 µM did not differ between primary (*n*=9) and recurrent GBMs (*n*=2). **(C)** However, when stratified for MGMT methylation status including recurrent tumors (*n*=11) or **(D)** without (*n*=9), only the topoisomerase inhibitor irinotecan demonstrated higher sensitivity in MGMT unmethylated GBMs (*P* = 0.02, Arrow). Unpaired Student’s t-test corrected for multiple comparisons (Holm-Sidak) was performed to calculate the *P*-value. *P*-values < 0.05 were considered significant (* *P* < .05). Data are presented as mean+SEM.

***
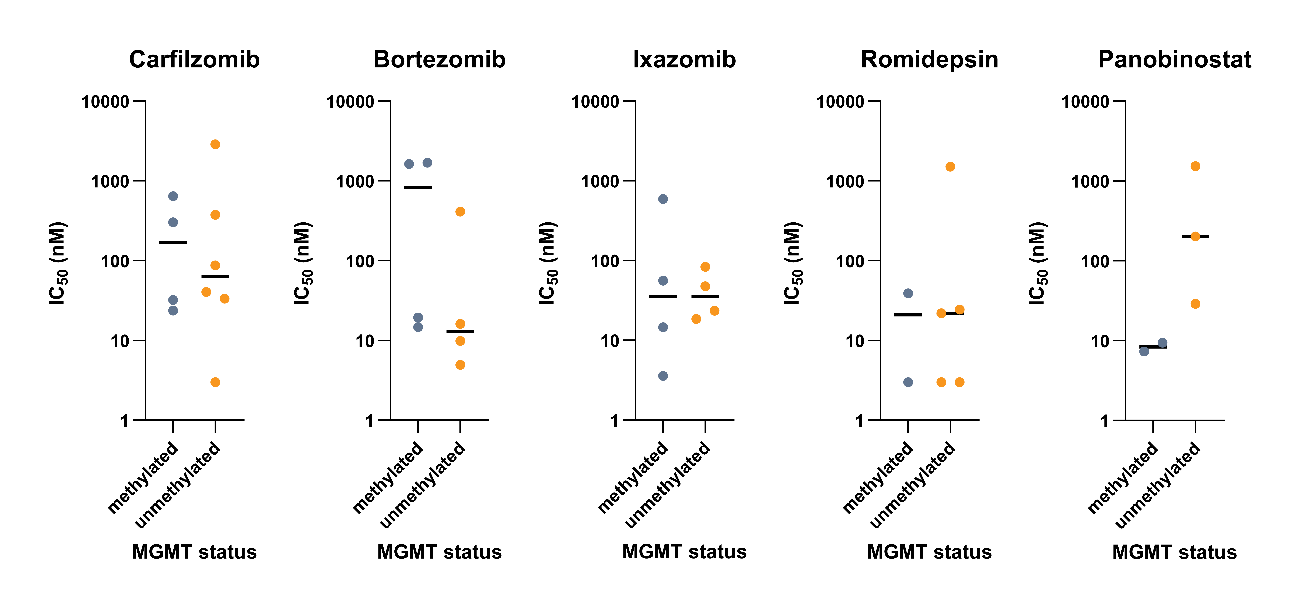
Supplementary Figure 2 – MGMT methylation status does not affect ex vivo drug sensitivity***

IC_50_ values for carfilzomib, bortezomib, ixazomib, romidepsin, and panobinostat were compared between MGMT-methylated and MGMT-unmethylated GBMs. Each dot represents one tumor sample; bars indicate the median. Statistical significance was assessed using the Mann-Whitney U test. No significant differences were observed between groups.


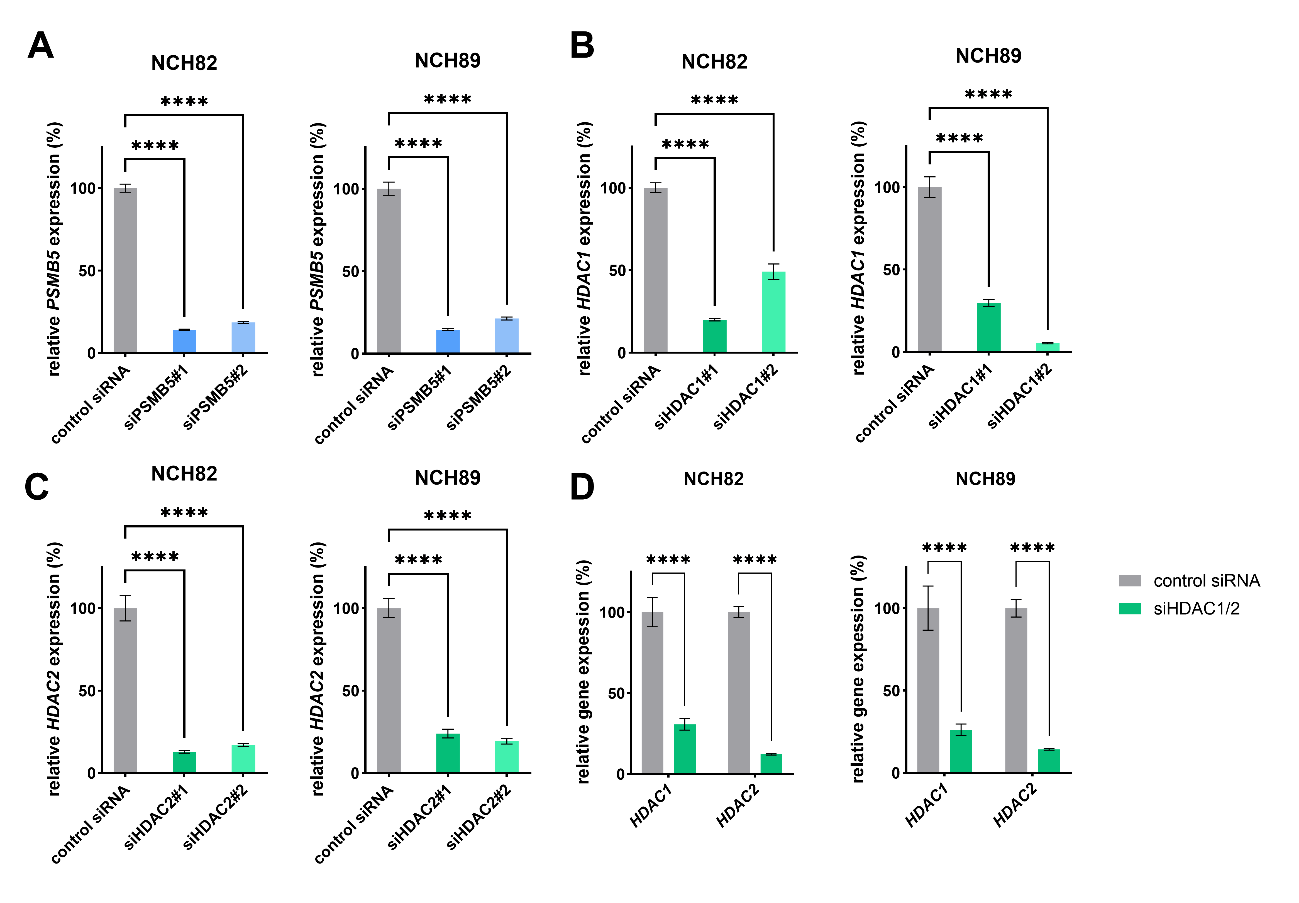


***Supplementary Figure 3 - Quantitative analysis of knockdown efficiencies for PSMB5, HDAC1, and HDAC2 in GBM cell lines using qRT-PCR***

Knockdown efficiencies for **(A)** *PSMB5*, **(B)** *HDAC1*, **(C)** *HDAC2*, and **(D)** *HDAC1/2* in NCH82 and NCH89 glioma cell lines were evaluated by quantitative real-time PCR. Data was normalized to control RNAi-transfected cells. Data are presented as mean+SEM. Dunnet’s one-way ANOVA was used to calculate *P*-values. *P*-values < 0.05 were considered significant (**** *P* < .0001).

**SUPPLEMENTARY TABLES**

**Suppl. Table S1:** List of IC_50_ data (nM) of top five tested drugs from each patient.

| **Nr** | **Primary or**  **Recurrent Tumor** | **MGMT status** | **Top 5 drugs** | | | | |
| --- | --- | --- | --- | --- | --- | --- | --- |
|  |  |  | **1** | **2** | **3** | **4** | **5** |
| 1 | Primary | methylated | NA | NA | NA | NA | NA |
| 2 | Primary | unmethylated | Carfilzomib  (2873.00) | Bortezomib  (410.90) | Romidepsin  (22.01) | Panobinostat  (1532.00) | Omacetaxine  (547.40) |
| 3 | Primary | unmethylated | Carfilzomib  (40.65) | Romidepsin  (3.00) | Panobinostat  (28.98) | Dactinomycin  (19.33) | Sorafenib  (>10000) |
| 4 | Recurrent | methylated | Carfilzomib  (642.70) | Bortezomib  (1633.00) | Ixazomib  (593.30) | Panobinostat  (>10000) | Idarubicin  (1875.00) |
| 5 | Primary | methylated | Carfilzomib  (303.80) | Bortezomib  (1695.00) | Ixazomib  (56.11) | Romidepsin  (38.92) | Epirubicin  (>10000) |
| 6 | Primary | unmethylated | Carfilzomib  (87.31) | Ixazomib  (23.52) | Romidepsin  (3.00) | Panobinostat  (202.40) | Lenalidomide  (>10000) |
| 7 | Primary | unmethylated | Carfilzomib  (33.38) | Bortezomib  (4.97) | Ixazomib  (83.61) | Omacetaxine  (2191.00) | Plicamycin  (1162.00) |
| 8 | Primary | unmethylated | Carfilzomib  (374.60) | Bortezomib  (9.85) | Ixazomib  (18.53) | Romidepsin  (24.39) | Alpelisib  (>10000) |
| 9 | Recurrent | methylated | Carfilzomib  (32.15) | Bortezomib  (19.49) | Ixazomib  (14.64) | Panobinostat  (9.43) | Idarubicin  (1731.00) |
| 10 | Primary | methylated | Carfilzomib  (23.75) | Bortezomib  (14.76) | Ixazomib  (3.58) | Romidepsin  (3.00) | Panobinostat  (7.32) |
| 11 | Primary | unmethylated | Carfilzomib  (3.00) | Bortezomib  (16.13) | Ixazomib  (47.69) | Romidepsin  (1509.00) | Lenalidomide  (>10000) |

| Gene |  | Sequence [5'->3'] |
| --- | --- | --- |
| *HDAC1* | forward | TTGTCTACTGGTGGTTCTGTGG |
|  | reverse | AGATGCCTCGGACTTCTTTG |
| *HDAC2* | forward | TGCTTGCCATCCTTGAATTA |
|  | reverse | CCGTCATTACACGATCTGTTG |
| *PSMB5* | forward | GATGGCTGGATCCGAGTCTC |
|  | reverse | TCTTTCAGGGGGTAGAGCCA |
| *ACTB* | forward | CCAACCGCGAGAAGATGA |
|  | reverse | CCAGAGGCGTACAGGGATAG |
| *HPRT1* | forward | TGACCTTGATTTATTTTGCATACC |
|  | reverse | CGAGCAAGACGTTCAGTCCT |
| *GAPDH* | forward | AGCCACATCGCTCAGACAC |
|  | reverse | GCCCAATACGACCAAATCC |

**Suppl. Table S2:** List of primers for qRT-PCR validation
